# Coexistence of Humans and Hamadryas Baboons in Al-Baha Region, Saudi Arabia—Emotional, Social, and Financial Aspects

**DOI:** 10.3390/ani16010047

**Published:** 2025-12-24

**Authors:** Salihah Alghamdi, Dietmar Zinner, Mansour AlMalki, Seham Salamah, Saleh Al-Ghamdi, Mohammed Althubyani, Abdullah Al-Ghamdi, Wael Alzahrani, Abdulaziz Alzahrani, Ghanem Al-Ghamdi

**Affiliations:** 1 Department of Mathematics, College of Science, Al-Baha University, Al-Baha 65799, Saudi Arabia; malthobyani@bu.edu.sa; 2 Cognitive Ecology Laboratory, German Primate Center, Leibniz Institute for Primate Research, 37077 Göttingen, Germany; dzinner@gwdg.de; 3 Department of Primate Cognition, Georg-August-Universität Göttingen, 37077 Göttingen, Germany; 4 Department of Sociology and Social Work, College of Arts and Humanities, King Abdulaziz University, Jeddah 21589, Saudi Arabia; mahalmalki1@kau.edu.sa (M.A.); shslamah@kau.edu.sa (S.S.); 5 Biology Department, College of Science, Al-Baha University, Al-Baha 65799, Saudi Arabia; sb.alghamdi@bu.edu.sa (S.A.-G.); ab.showail@gmail.com (A.A.-G.); wail1_2008@hotmail.com (W.A.); galghamdi@bu.edu.sa (G.A.-G.); 6 Department of Architecture, College of Engineering, Al-Baha University, Al-Baha 65799, Saudi Arabia; azahran@bu.edu.sa

**Keywords:** baboon impact, human–baboon interaction, Saudi Arabia, semi-structured online interviews

## Abstract

Human–wildlife conflicts arise from increasing human populations and the growing demand for land for agriculture and urban development. In Saudi Arabia, these dynamics have increased the impact of baboons on human communities, as expanding settlements encroach upon the natural habitats of baboons, while rising baboon populations increasingly invade urban areas in search of food, shelter, and water. We aimed to assess the effects of human–baboon coexistence on residents in the Al-Baha region, Saudi Arabia. From October 2021 to April 2022, we administered a 43-item semi-structured online questionnaire addressing emotional, social, environmental and financial impacts of nearby baboons. A total of 318 residents of the Al-Baha region completed the survey and shared their experiences regarding interactions with hamadryas baboons (*Papio hamadryas*) near human properties. In addition, three semi-structured life interviews with residents aged over 70 explored historical perceptions. Respondents attributed increased baboon presence to urban expansion, accessible waste, and intentional feeding and strongly supported government-led mitigation plans. Human–baboon interactions in Al Baha produce emotional stress, social disruption, and economic burdens for residents. Findings support integrated management combining public education, improved waste management, non-lethal deterrents, and carefully planned population control measures, developed with community consultation. Limitations of the study include convenience online sampling and reliance on self-reported impacts; future work should quantify baboon abundance and objectively measure economic losses.

## Simple Summary

Human–baboon conflicts are increasing in the Al-Baha region of Saudi Arabia as urban development expands into natural baboon habitats and baboon populations grow. These interactions affect residents emotionally, socially, and economically, as baboons enter urban areas in search of food, water, and shelter. To understand these impacts, we conducted an online survey with 318 residents and in-depth interviews with three older participants, exploring experiences with hamadryas baboons. Residents reported that urban expansion, accessible waste, and intentional feeding contributed to more frequent baboon encounters, and they strongly support government-led mitigation strategies. Our findings highlight the need for integrated management approaches that combine public education, improved waste handling, non-lethal deterrents, and carefully planned population control, developed with community involvement. These results provide practical guidance for reducing human–baboon conflicts while promoting coexistence.

## 1. Introduction

The persistent growth of the human population has increasingly pressured natural habitats and the resources available to wildlife [1]. Human–wildlife conflict, defined by the 2003 World Park Congress [2] as occurring when wildlife requirements overlap with human needs, producing costs for humans and animals, now commonly arises in urban and peri-urban areas as well as agricultural landscapes [3,4]. Urbanization often degrades and fragments natural habitats [5], while some species adapt to and exploit urban environments [6].

In Africa, baboons (*Papio*) and green monkeys (*Chlorocebus*) frequently feature among crop and property pests [7,8,9,10,11]. In particular, baboons can cause significant loss in agriculture and forestry [12,13,14,15,16,17]. Similarly, in South and South-East Asia, macaques (*Macaca*) cause substantial agricultural and infrastructure damage [18,19,20]. Due to their religious role, macaques and hanuman langurs (*Semnopithecus entellus*) have been tolerated and living around temples and in cities for centuries [21].

The emergence of baboons as a problem in urban and peri-urban areas has been observed primarily in the last few decades, notably in Southern Africa and Saudi Arabia [22,23,24,25,26].

A critical aspect of human–non-human primate conflicts is the perception and attitude of local residents toward these species. Gathering public opinion through interviews, surveys, or other methods is essential for informing effective mitigation strategies [27,28,29,30,31,32].

In Saudi Arabia, hamadryas baboons (*Papio hamadryas*) are the only naturally occurring non-human primates [33]. Until approximately 50 years ago, hamadryas baboons in the Arabian Peninsula were primarily considered agricultural pests for their crop raiding [34]. Since the 1960s, Saudi Arabia has undergone rapid economic development, accompanied by a substantial increase in the human population—from 7 million in 1974 to 34 million in 2021 [35]. This growth, besides urban expansion, has encroached on traditional baboon habitats. Additionally, the abundance of human-derived food and the extirpation of natural predators, such as the Arabian leopard (*Panthera pardus nimr*) [36], likely contributed to increasing baboon populations in the region.

Al-Baha region is the smallest governorate in Saudi Arabia (~11,000 km^2^; 20°00′45″ N 41°27′55″ E), with an elevation from 143 to 2155 m above sea level [37]. Its population resides across 10 main cities and nearly 1200 villages. The economy includes government employment, small businesses, trade, tourism, and, to a minor extent, farming. Land cover consists of urban development, bare soil, dry savannah, and irrigated and non-irrigated agriculture [38]. Over the past three decades, urbanization has transformed significant portions of Al-Baha’s natural habitat, reducing the historical habitat of baboons. The urban areas in Al-Baha region expanded from only 15 km^2^ (0.13%) in 1985 to 1982 km^2^ (17.85%) in 2021, mainly at the expense of rangeland, forest, and shrubland [39]. At the same time, the baboons had access to new food sources due to the massive volumes of waste produced, including leftover human food, in enormous open rubbish dumps and waste containers. Additionally, feeding baboons became a recreational activity for the public, giving them high-quality food that probably enhanced their rate of reproduction [22].

The close coexistence of humans and baboons in urban and peri-urban areas has generated conflicts, including infrastructure damage, house and garden raids, contamination of children’s playgrounds and public parks, and, in some cases, attacks on humans. These interactions also raise public health concerns, as several cases in baboons have reported gastrointestinal parasites, bacteria, and viruses [40,41,42,43,44,45].

To assess local awareness of these issues and support mitigation efforts, we conducted an online questionnaire survey. This study aimed to identify the emotional, social, and financial impacts of baboons on residents’ lives and examine residents’ attitudes toward baboons. The findings are intended to inform policymakers in developing strategies to manage human–baboon coexistence in the Al-Baha region.

## 2. Methods

### 2.1. Study Area

The study was conducted in Al-Baha, located in the southwest of Saudi Arabia. The region is characterized by high mountains reaching up to 2000 m and a high-altitude plateau with steep cliffs, particularly along its western edge. Situated in the mountainous area along the Red Sea, Al-Baha city lies at an elevation of approximately 1900 m above sea level. The region experiences a generally warm weather, with temperatures ranging from 7 °C to 32 °C and annual precipitation between 150 and 200 mm [46]. The pleasant climate and scenic mountainous landscape make Al-Baha a desirable place to live and one of the Kingdom’s prime tourist destinations. In 2022, the population of the Al-Baha region was estimated at over 339,000 residents [47].

Hamadryas baboons are present throughout all districts of Al-Baha, with their primary distribution concentrated along the western mountain range extending from northwest to southeast. They are frequently observed in Al-Baha city center, smaller towns and villages, school areas, commercial zones, waste management facilities, regional recreational parks, and the remaining natural habitats [25].

### 2.2. Data Collection

We used an online questionnaire survey to gather information on the emotional, social, environmental, and financial effects (four dimensions) of baboons on Al-Baha people between 17 October 2021, and 10 April 2022. (see questionnaire in the Supplementary Materials). The target participants were residents living in areas with potential human–baboon conflicts. Participation was voluntary. The questionnaire was made accessible in October 2021, following an announcement of the study on social media groups two weeks before its release.

The questionnaire consisted of 43 questions and required approximately 20–30 min to complete. Both quantitative and qualitative items were included, addressing four key aspects: emotional impact, social impact, environmental impact, and financial impact. A total of 318 responses were received, all of which were included in the analyses.

The survey collected demographic information, participants’ perceptions and feelings toward baboons residing nearby, attitudes toward the species, and opinions regarding the impact of baboons on the natural environment. Participants also provided information on financial losses resulting from baboon activities on farms and properties, as well as their perspectives on potential management and mitigation strategies. Respondent confidentiality was maintained by replacing individual identifiers with numeric codes.

### 2.3. Selected Interviews

In addition to the survey, in-depth interviews were conducted with three individuals over 70 years of age. These interviews aimed to gather historical insights into human–baboon interactions in the region. Participants were asked about their historical understanding of baboon habitat, abundance, and whether or not baboon numbers have grown over time. They were also asked if they believe that the baboon population’s expansion was influenced by the past behavior and customs of the local population.

### 2.4. Statistical Analysis

We analyzed the responses descriptively and presented percentages on a three-level Likert scale (‘Disagree’, ‘Somewhat agree’, ‘Agree’). All completed questionnaires (*n* = 318) were included. Several items allowed multiple responses (e.g., types of damaged property); in such cases, percentages may sum to >100%. We used R software (R version 4.4.2) for all analyses. Cronbach’s alpha (α) was used to assess the reliability and internal consistency of the questionnaire items. We tested for gender differences in their response by applying Mann–Whitney tests. This non-parametric test was chosen due to the ordinal nature of the Likert-scale data and the non-normal distribution of responses. And we used Kruskal–Wallis tests when our predictor variable had more than two categories (e.g., Village, Small town, Big city).

## 3. Results

### 3.1. Respondents’ Characteristics

A total of 318 participants (Table 1) voluntarily responded to the survey over the seven-month study period, reporting their perceptions and attitudes toward baboons. The gender distribution indicated that 70% of respondents were male and 30% were female, spanning various age groups. Specifically, 28% were 30 years or younger, 25% were between 31 and 40 years, 27% were between 41 and 50 years, and 20% were over 50 years old.

Regarding educational background, 81% of respondents held a college degree. Employment status revealed that 51% were government employees, while 31% were unemployed. In terms of residence, nearly 50% lived in villages, followed by approximately 20% residing in the main cities. Farmers represented the largest group among respondents experiencing damage due to baboon incursions.

We did not find any differences among the responses of females and males in the four dimensions (emotional *n* = 318, U = 6214, *p* = 0.835; social *n* = 318, U = 5474, *p* = 0.418; environmental *n* = 318, U = 4802, *p* = 0.086; financial *n* = 318, U = 5538, *p* = 0.427), indicating that perceptions and experiences of human–baboon interactions were largely consistent across genders.

We also did not find differences in responses according to the different housing categories for the four dimensions (emotional *n* = 318, H = 6.41, *p* = 0.093; social *n* = 318, H = 6.72, *p* = 0.082; environmental *n* = 318, H = 6.32, *p* = 0.097; financial *n* = 318, H = 7.19, *p* = 0.066). These results indicate that participants’ perceptions and experiences of human–baboon interactions were largely similar across the various residential categories.

Similarly, no significant differences were detected in participants’ responses according to housing category across the four assessed dimensions (emotional *n* = 318, H = 7.90, *p* = 0.095; social *n* = 318, H = 3.69, *p* = 0.449; environmental *n* = 318, H = 3.32, *p* = 0.506; financial *n* = 318, H = 4.88, *p* = 0.300). Likewise, variations in education level did not produce statistically significant differences in any of the dimensions examined, suggesting that participants’ perceptions and experiences of human–baboon interactions were broadly consistent across both residential and educational backgrounds.

### 3.2. Responses According to the Four Dimensions

Cronbach’s alpha coefficients are equal to or above 0.70, suggesting acceptable to good reliability (emotional α = 0.72; social α = 0.80; environmental α = 0.70; financial α = 0.76).

#### 3.2.1. Emotional Aspect

**Figure 1:**
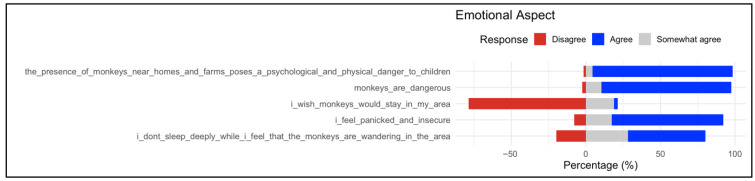
Distribution of responses on emotional aspects associated with the presence of baboons. The respective statements are listed on the left; proportions (%) of responses on the right as stacked bars (red = Disagree, gray = Somewhat agree, and blue = Agree). Responses revealed that the presence of monkeys is widely associated with psychological distress.

Analysis of the emotional dimension indicates consistent agreement among respondents regarding the perceived risks associated with baboons. As shown in Figure 1, the majority of participants agreed that “the presence of monkeys near homes and farms poses a psychological and physical danger to children” and that “monkeys are dangerous”, with over 75% selecting the “Agree” option. Similarly, high levels of agreement were observed for feelings of panic and insecurity, as well as difficulties in sleeping due to the presence of baboons. In contrast, a clear divergence was observed in the statement “I wish monkeys would stay in my area”, where disagreement was prevalent, suggesting that most respondents view the continued presence of monkeys in residential environments as unwelcome. Overall, these results highlight a strong perception of baboons as a source of emotional distress, with responses concentrated on agreement with negative emotional impacts and rejection of coexistence.

#### 3.2.2. Social Aspect

**Figure 2:**
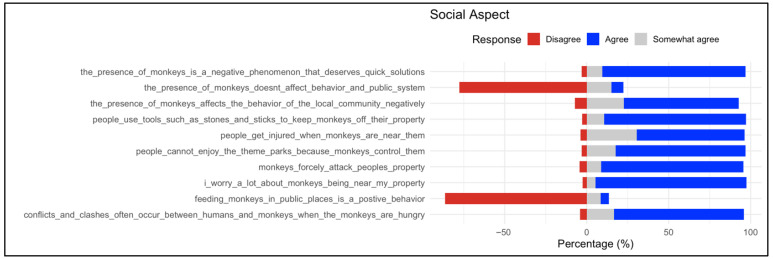
Distribution of responses on social aspects associated with the presence of baboons. The respective statements are listed on the left; proportions (%) of responses on the right as stacked bars (red = Disagree, gray = Somewhat agree, and blue = Agree). The result indicates that perceptions of the social impacts of monkeys were negative.

Responses regarding the social dimension revealed strong perceptions of negative community impacts associated with monkeys (Figure 2). The majority of participants perceived the presence of baboons as a negative phenomenon requiring prompt intervention and reported that baboons adversely affect the behavior of the local community. Seventy-nine percent indicated that conflicts and clashes with baboons frequently occur, particularly when the animals are hungry. Most respondents (87%) reported that baboons damage personal property, and 92% expressed concern about baboons being near their homes or belongings.

Participants also noted that recreational activities, such as visiting theme parks, were negatively impacted by the presence of baboons. A majority (86%) considered feeding baboons a harmful practice. While over 85% agreed that using tools such as stones or sticks can help keep baboons away from property, 66% acknowledged the risk of injury when encountering baboons. In contrast, disagreement predominated for the statement “the presence of monkeys does not affect behavior and public system” and for “feeding monkeys in public places is a positive behavior”, underscoring the recognition of monkeys as a social burden rather than a neutral or beneficial presence. All of these findings point to a generalized concern about the social consequences of baboons’ presence, with the majority of respondents focusing on negative community-level impacts.

#### 3.2.3. Environmental Aspect

**Figure 3:**
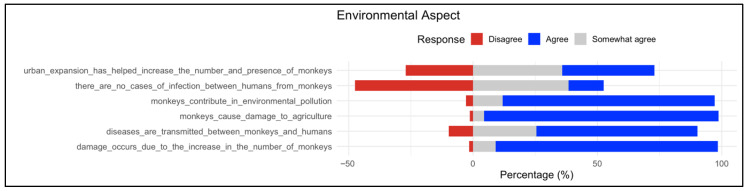
Distribution of responses on environmental aspects associated with the presence of baboons. The respective statements are listed on the left; proportions (%) of responses on the right as stacked bars (red = Disagree, gray = Somewhat agree, and blue = Agree). The findings underscore that financial losses due to monkey presence are widely recognized across the surveyed population.

Findings for the environmental dimension indicate strong public recognition of the ecological risks associated with monkeys (Figure 3). The majority of respondents agreed that monkeys contribute to environmental pollution, cause agricultural damage, and that damage increases with the rise in monkey numbers. High levels of agreement were also observed for the statement that diseases are transmitted between monkeys and humans, underscoring public awareness of infections and diseases that can be spread between people and animals. Conversely, disagreement predominated for the claim that “there are no cases of infection between humans from monkeys”, further reinforcing perceptions of disease transmission as a serious concern. Similarly, most respondents agreed that urban expansion has led to an increase in monkey populations, suggesting that human-driven landscape change is perceived as a contributing factor. All of these findings point to widespread agreement that monkeys have negative effects on the environment through ecological, agricultural, and health-related pathways.

#### 3.2.4. Financial Aspect

**Figure 4:**
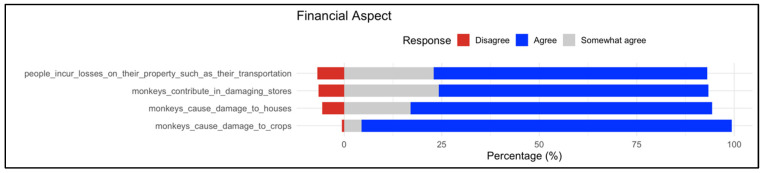
Distribution of responses on financial aspects associated with the presence of baboons. The respective statements are listed on the left; proportions (%) of responses on the right as stacked bars (red = Disagree, gray = Somewhat agree, and blue = Agree). The results indicate that monkeys contribute to substantial economic losses.

The financial impacts of baboons on properties, including homes, farms, and other businesses, were assessed. The majority of participants agreed that baboons impose significant financial pressure on residents of the region (Figure 4). In particular, 70% of respondents said they had lost property, including farming vehicles and transportation tools. Over 77% indicated that baboons caused damage to houses, while 69% reported damage to business establishments. Notably, more than 95% of respondents experienced damage to their farming properties.

With respect to damage frequency, the majority of participants (72%) reported experiencing property damage on a weekly basis, while 17% indicated monthly incidents and 11% reported damage occurring annually. In terms of financial burden, 23% of respondents reported losses exceeding 20,000 Saudi Riyals (≈USD 5000), 25% reported losses ranging between 10,000–20,000 SR (≈USD 2500–5000), and the remaining 52% experienced damages amounting to less than 10,000 SR (≈USD 2500). These findings highlight that both the frequency and financial severity of monkey-related damages are substantial for affected households.

#### 3.2.5. Suggested Solutions to Control the Presence of Baboons

**Figure 5:**
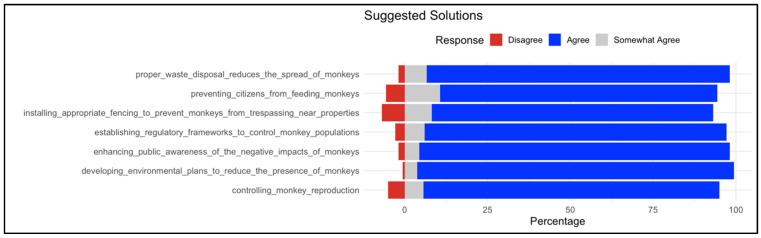
Distribution of responses on suggested solution to the ‘baboon problem’. The respective statements are listed on the left; proportions (%) of responses on the right as stacked bars (red = Disagree, gray = Somewhat agree, and green = Agree). Diverging stacked bar chart showing participant agreement with proposed solutions for mitigating baboon impacts. Responses were measured.

Over 84% of participants support the proposed strategies for managing and limiting baboon populations (Figure 5). These measures ranged from relatively soft interventions, such as raising public awareness, to more direct approaches, including population control. Among the options presented, the highest level of agreement was observed for the implementation of a comprehensive environmental management plan by governmental authorities.

### 3.3. Selected Interviews

Three participants over 70 years of age were interviewed to gain insights into the historical extent of human–baboon conflicts. According to their accounts, baboons historically inhabited cliffs in the mountains of Al-Baha in limited numbers, likely not exceeding 15,000 individuals. Most frequently, groups of fewer than 100 people were observed. The respondents noted that natural predators and hunters historically helped maintain low baboon populations. However, in recent years, their numbers have grown significantly, almost reaching five times their previous population size. The interviewees largely ascribed this increase to human activity, specifically the supply of substantial amounts of food derived from humans, whether intentional or inadvertent.

## 4. Discussion

The increasing baboon population in the Al-Baha region, their wide distribution, and frequent incursions into villages and towns in search of food, shelter, and water have significant impacts on local residents [25]. To investigate residents’ experiences with baboons, we conducted an online survey via social media over a seven-month period. Most respondents were adult, well-educated males aged 30–50, employed primarily by the government. Many had lived in the area for years and had extensive experience with baboon presence. Given that the government frequently paid these respondents a stable yearly salary and that they also engage in some farming, their financial losses caused by baboons did not appear to threaten their overall livelihood.

Residents reported multiple forms of conflict with baboons that affected their daily lives. The majority of participants expressed concern for the safety of children, considering baboons as both psychologically stressful and physically threatening. Similar concerns have been reported in Zimbabwe, where aggressive baboons posed risks to children attending school [48]. Large baboon groups can provoke fear and panic, especially when residents attempt to protect property, potentially escalating confrontations.

Common mitigation strategies included high walls surrounding the house, securing windows and doors, and sometimes raising fencing with electric wires. However, the more likely course of action for self-defense and/or preventing a baboon assault was to take rapid action, such as stoning and using sticks. Systematic property guarding, although effective in other countries [49], is not widely practiced in Al-Baha. Locals supported more comprehensive measures such as enforcing national policies to address human–baboon conflicts, raising public awareness, and regulating human–baboon interactions. In African and Asian contexts, family-based guarding (women and children as well as men), physical barriers, alarms, and repellents are commonly used to protect farmland [49,50]. However, other precautions such as an alert system, reducing field guard shifts, having male guards, and having enough guards during the peak of the baboons’ presence of monkeys are necessary for effective guarding [49]. The impact of baboons is substantial, despite the efforts of the locals in Al-Baha to reduce such harm.

Social impacts have been evident in areas where humans and baboons come into conflict. While most people recognize that the primary driver of these encounters is the baboons’ search for food, this behavior poses challenges for families seeking to engage in outdoor leisure activities in regional parks, near residential areas, or even during routine grocery shopping. Outdoor cooking and food preparation, a main activity of social gatherings, simultaneously serve as major attractants drawing baboons closer to human spaces.

Consequently, instead of enjoying recreational activities, many families redirect their efforts toward protective behaviors, such as monitoring children and personal belongings (e.g., food, cookware), or resorting to deterrence strategies like throwing stones or using available objects to repel baboons. Notably, a substantial proportion of individuals also expressed concern about the risk of physical injury associated with such encounters.

The ecological consequences of village development and urban expansion are often underestimated. A considerable proportion of people remain unaware of the importance of conserving wildlife within its natural habitats. The proximity of human settlements to wildlife zones increases the likelihood, both in frequency and scale, of baboon incursions into residential areas. Similar patterns of baboon behavior have been documented in South Asia, where the extent of human–baboon interactions was also influenced by the type of cultivated crops [19].

In this study, the relationship between specific crop types and their susceptibility to baboon foraging was not examined. However, incidents occurring along the interface between wildlife habitats and human properties were closely assessed, highlighting an area that warrants further investigation. Previous research focusing on the contact zones between farmland and wildlife has reported elevated risks of conflict [12,19]. Despite the relatively high educational attainment among participants—most of whom held university degrees—there remains a pressing need for enhanced awareness regarding the importance of conserving natural ecosystems for future generations.

Interestingly, while earlier assumptions suggested that local communities possessed a limited understanding of wildlife conservation, our findings reveal that negative experiences with baboons, particularly concerning food-related resources (e.g., farms, restaurants, kitchens, groceries), may shape perceptions. Participants expressed concern about the role of baboons in the transmission of infectious diseases, which underscores a heightened awareness of public health risks. This suggests that rather than a lack of knowledge, attitudes may reflect the tangible impacts of baboon-related damages and the perceived threat of zoonotic disease transmission through direct or indirect contact.

Financial impacts were also reported by the examined population in areas where conflicts between humans and baboons occur. Conflicts resulted in damage to houses, vehicles, farms, crops, and business properties. Although the majority of residents are employed in government sectors or operate businesses not entirely dependent on farming income, such damages nonetheless impose a noticeable financial burden. Crop raiding, as well as damage to farming equipment and tools, were common concerns, and households, businesses, and other assets were also affected. Most participants estimated losses of less than SR10,000 (≈USD 2500); however, some reported damages exceeding SR20,000 (≈USD 5000). These costs are considerably higher than those documented in comparable studies from Asian countries [19]. Such financial losses were reported to occur on a near-weekly basis. Nevertheless, the severity of their long-term financial impact appears to be mitigated by the presence of alternative household income sources. Despite this, the recurrent nature of the damages places families under continuous financial and psychological pressure, requiring constant vigilance to prevent or address losses.

Historical interviews with residents over 70 years old, who spent their time in locations where they had direct contact with baboons, indicated that baboon populations were previously smaller and kept in check by natural predators and hunters. Access to human-derived food was limited, maintaining a more balanced coexistence and healthy environment for baboons and humans alike. In contrast, population growth, urban expansion, habitat loss, and abundant food resources have allowed baboons to increase in number, become more tolerant of humans, and exhibit more aggressive behaviors. Residents emphasized the urgent need for interventions to minimize the negative impacts of baboons on residential, farming, and business areas. While immediate solutions are required to address conflicts, participants also highlighted the importance of a comprehensive environmental management plan. Local and regional authorities are reportedly working toward such strategies, and this study may provide valuable guidance for decision-makers [51].

## 5. Conclusions

The growing baboon population in the Al-Baha region has resulted in notable social, environmental, and economic challenges for local communities. Urban encroachment on baboon habitats drives these animals into human settlements, increasing the frequency of conflicts. Residents expressed concerns about emotional and physical risks, particularly for children and families engaged in outdoor activities. Despite efforts to implement physical barriers, baboon incursions continue to disrupt daily life, necessitating constant vigilance to protect property. While most residents have stable incomes, the cumulative effects of property damage, crop losses, and repair costs impose a significant burden.

The findings underscore the need for increased wildlife conservation awareness and the development of a comprehensive management plan to mitigate human–baboon conflicts. Collaborative efforts between local authorities and communities are essential to implement long-term, balanced solutions to ensure a balanced coexistence between humans and wildlife.

**Future Directions:** While this study provides important insights into human–baboon interactions in the Al-Baha Region, several avenues remain for further investigation. Comparative studies across regions with similar baboon populations, such as Asir or Taif, could help identify context-specific versus generalizable patterns of human–baboon conflict. Investigating baboon behavior and ecology, including troop dynamics, foraging patterns, and seasonal movements, would further illuminate why certain areas experience more intense interactions. Future research could also examine the ecological and behavioral factors that contribute to baboon intrusions, including the types of crops and food sources that attract baboons most. Longitudinal studies assessing changes in baboon population dynamics and human adaptation strategies over time would provide a more comprehensive understanding of conflict trends. Research should also explore intergenerational perceptions of baboons and human–wildlife interactions. Understanding how knowledge, attitudes, and experiences differ across age groups can inform education and awareness programs tailored to foster coexistence. Analyzing the coping measures used by impacted communities, from social and cultural strategies to physical barriers and guarding behaviors, can also reveal areas that require more support and highlight successful local solutions.

## Figures and Tables

**Table 1:** Demographic and socioeconomic traits of participants.

Variable	Category	Number of Respondents
Gender	Female	94 (30%)
	Male	224 (70%)
Age	<30	89 (28%)
	30–39	81 (25%)
	40–49	84 (27%)
	>50	64 (20%)
Education	None	1 (0.3%)
	Primary	1 (0.3%)
	Intermediate	4 (1.3%)
	Secondary	53 (16.7%)
	High education	259 (81.4%)
Employment	Unemployed	100 (31%)
	Government employee	164 (51%)
	Private sector employee	38 (12%)
	Farmer	8 (3%)
	Seller	8 (3%)
Housing geography	Big city	66 (21%)
	Medium city	49 (15%)
	Small town	53 (17%)
	Village	150 (47%)
Damaged property	Farm	153 (48%)
	Residence	95 (30%)
	Store	2 (0.6%)
	Others	68 (21.4%)

## Data Availability

The data presented in this study are available on request from the corresponding author due to participant confidentiality and ethical restrictions.

## Supplementary Materials

The following supporting information can be downloaded at: https://www.mdpi.com/article/10.3390/ani16010047/s1, Questionnaire: The survey used in this study to collect data from participants. The questionnaire includes five main sections: (1) demographic information, (2) emotional aspect, (3) social aspect, (4) environmental aspect, and (5) financial aspect. All items were presented using a structured format with closed-ended questions and Likert-type scales.

## References

[1] Cowlishaw G., Dunbar R.I.M. (2000). Primate Conservation Biology.

[2] IUCN World Park Congress (WPC) (2003). Preventing & Mitigating Human–Wildlife Conflicts.

[3] Soulsbury C.D., White P.C.L. (2016). Human–wildlife interactions in urban areas: A review of conflicts, benefits and opportunities. Wildl. Res..

[4] Zimmermann A., McQuinn B., Macdonald D.W. (2020). Levels of conflict over wildlife: Understanding and addressing the right problem. Conserv. Sci. Pract..

[5] Simkin R.D., Seto K.C., McDonald R.I., Jetz W. (2022). Biodiversity impacts and conservation implications of urban land expansion projected to 2050. Proc. Natl. Acad. Sci. USA.

[6] Schilthuizen M. (2018). Darwin Comes to Town: How the Urban Jungle Drives Evolution.

[7] Else J.G., Box H.O. (1991). Nonhuman primates as pests. Primate Responses to Environmental Change.

[8] Strum S.C. (1994). Prospects for management of primate pests. Rev. Écol..

[9] Strum S.C. (2010). The development of primate raiding: Implications for management and conservation. Int. J. Primatol..

[10] Hill C.M. (2000). Conflict of interest between people and baboons: Crop raiding in Uganda. Int. J. Primatol..

[11] Hill C.M. (2018). Crop foraging, crop losses, and crop raiding. Annu. Rev. Anthropol..

[12] Naughton-Treves L. (1997). Farming the forest edge: Vulnerable places and people around Kibale National Park, Uganda. Geogr. Rev..

[13] Kagoro-Rugunda G. (2004). Crop raiding around Lake Mburo National Park. Afr. J. Ecol..

[14] Katsvanga C.A.T., Jimu L., Mupangwa J.F., Zinner D. (2009). Susceptibility of pine stands to bark stripping by chacma Papio ursinus baboons in the Eastern Highlands of Zimbabwe. Curr. Zool..

[15] Findlay L.J. (2016). Human-Primate Conflict: An Interdisciplinary Evaluation of Wildlife Crop Raiding on Commercial Crop Farms in Limpopo Province, South Africa. Ph.D. Thesis.

[16] Kifle Z., Bekele A. (2021). Human–hamadryas baboon (*Papio hamadryas*) conflict in the Wonchit Valley, South Wollo, Ethiopia. Afr. J. Ecol..

[17] Jaleta M., Tekalign W. (2023). Crop loss and damage by primate species in Southwest Ethiopia. Int. J. Ecol..

[18] Sekhar N.U. (1998). Crop and livestock depredation caused by wild animals in protected areas: The case of Sariska Tiger Reserve, Rajasthan, India. Environ. Conserv..

[19] Koirala S., Garber P.A., Somasundaram D., Katuwal H.B., Ren B., Huang C., Li M. (2021). Factors affecting the crop raiding behavior of wild rhesus macaques in Nepal: Implications for wildlife management. J. Environ. Manag..

[20] Rudran R., Cabral de Mel S.J., Sumanapala A., De Mel R.K., Mahindarathna K.K.T. (2021). An ethnoprimatological approach to mitigating Sri Lanka’s human–monkey conflicts. Primate Conserv..

[21] Priston N.E.C., McLennan M.R., Radhakrishna S., Huffman M.A., Sinha A. (2013). Managing humans, managing macaques: Human–macaque conflict in Asia and Africa. The Macaque Connection: Cooperation and Conflict Between Humans and Macaques.

[22] Biquand S., Boug A., Biquand-Guyot V., Gautier J.P. (1994). Management of commensal baboons in Saudi Arabia. Rev. Écol..

[23] Hoffman T.S., O’Riain M.J. (2012). Monkey management: Using spatial ecology to understand the extent and severity of human–baboon conflict in the Cape Peninsula, South Africa. Ecol. Soc..

[24] Fehlmann G., O’Riain M.J., Fürtbauer I., King A.J. (2021). Behavioral causes, ecological consequences, and management challenges associated with wildlife foraging in human-modified landscapes. BioScience.

[25] Al-Ghamdi G., Alzahrani A., Al-Ghamdi S., Alghamdi S., Al-Ghamdi A., Alzahrani W., Zinner D. (2023). Potential hotspots of hamadryas baboon–human conflict in Al-Baha Region, Saudi Arabia. Diversity.

[26] Mazué F., Guerbois C., Fritz H., Rebout N., Petit O. (2023). Less bins, less baboons: Reducing access to anthropogenic food effectively decreases the urban foraging behavior of a troop of chacma baboons (*Papio hamadryas ursinus*) in a peri-urban area. Primates.

[27] Knight J., Hill C.M., Webber A.D., Priston N.E.C. (2017). Block, push or pull? Three responses to monkey crop-raiding in Japan. Understanding Conflicts About Wildlife: A Biosocial Approach.

[28] Cabral S.J., Prasad T., Deeyagoda T.P., Weerakkody S.N., Nadarajah A., Rudran R. (2018). Investigating Sri Lanka’s human–monkey conflict and developing a strategy to mitigate the problem. J. Threat. Taxa.

[29] Dittus W.P.J., Gunathilake S., Felder M. (2019). Assessing public perceptions and solutions to human–monkey conflict from 50 years in Sri Lanka. Folia Primatol..

[30] Pebsworth P.A., Radhakrishna S. (2021). The costs and benefits of coexistence: What determines people’s willingness to live near nonhuman primates?. Am. J. Primatol..

[31] Karimullah K., Widdig A., Sah S., Amici F. (2022). Understanding potential conflicts between human and non-human primates: A large-scale survey in Malaysia. Biodivers. Conserv..

[32] Poornima A.M.N.S., Weerasekara W.M.L.S., Vinobaba M., Karunarathna K.A.N.K. (2022). Community-level awareness and attitudes towards human–monkey conflict in Polonnaruwa district, Sri Lanka. Primates.

[33] Harrison D.L. (1964). The Mammals of Arabia, Vol. I. Insectivora, Chiroptera, Primates.

[34] Harrison D.L. (1968). The large mammals in Arabia. Oryx.

[35] Saudi General Authority for Statistics (GASTAT) (2021). Statistical Yearbook of 2021.

[36] Dunford C., Faure J., Ross M., Spalton J., Drouilly M., Pryce-Fitchen K., Mann G. (2024). Searching for spots: A comprehensive survey for the Arabian leopard Panthera pardus nimr in Saudi Arabia. Oryx.

[37] (2019). HABITAT City Prosperity Index: Al-Baha. United Nations Saudi Arabia. https://saudiarabia.un.org/en/40072-city-prosperity-index-albaha.

[38] Mahmoud S.H., Alazba A.A. (2016). Land cover change dynamics mapping and predictions using EO data and a GIS-cellular automata model: The case of Al-Baha region, Kingdom of Saudi Arabia. Arab. J. Geosci..

[39] Alsharif M., Alzandi A.A., Shrahily R., Mobarak B. (2022). Land use land cover change analysis for urban growth prediction using Landsat satellite data and Markov Chain Model for Al-Baha Region Saudi Arabia. Forests.

[40] Nasher A.K.A. (1988). Zoonotic parasite infections of the Arabian sacred baboon Papio hamadryas arabicus Thomas in Asir Province, Saudi Arabia. Ann. Parasitol. Hum. Comp..

[41] Ghandour A.M., Zahid N.Z., Banaja A.A., Kamal K.B., Bouq A.I. (1995). Zoonotic intestinal parasites of hamadryas baboons Papio hamadryas in the western and northern regions of Saudi Arabia. J. Trop. Med. Hyg..

[42] Zahed N.Z., Ghandour A.M., Banaja A.A., Banerjee R.K., Dehlawi M.S. (1996). Hamadryas baboons Papio hamadryas as maintenance hosts of Schistosoma mansoni in Saudi Arabia. Trop. Med. Int. Health.

[43] Alqumber M.A. (2014). Association between Papio hamadryas populations and human gastrointestinal infectious diseases in southwestern Saudi Arabia. Ann. Saudi Med..

[44] Olarinmoye A.O., Olugasa B.O., Niphuis H., Herwijnen R.V., Verschoor E., Boug A., Ishola O.O., Buitendijk H., Fagrouch Z., Al-Hezaimi K. (2017). Serological evidence of coronavirus infections in native hamadryas baboons (*Papio hamadryas hamadryas*) of the Kingdom of Saudi Arabia. Epidemiol. Infect..

[45] Kasem S., Hussein R., Al-Doweriej A., Qasim I., Abu-Obeida A., Almulhim I., Alfarhan H., Hodhod A.A., Abel-latif M., Hashim O. (2019). Rabies among animals in Saudi Arabia. J. Infect. Public Health.

[46] Climate and Average Weather Year Round in Al Bahah, Saudi Arabia. WeatherSpark. https://weatherspark.com/y/101924/Average-Weather-in-Al-Bahah-Saudi-Arabia-Year-Round.

[47] Saudi Census (2022). General Authority of Statistics. https://www.stats.gov.sa/en/.

[48] Marisa L., Chimwe M., Zhou K., Chinofunga A.T., Njini B., Mugadza L., Nkomo P., Dirwayi T.P. (2022). Baboon–human conflict (BHC), an emerging urban crisis faced by residents in Redcliff Municipality, Zimbabwe. Glob. Sci. J..

[49] Findlay L.J., Hill R.A. (2020). Field guarding as a crop protection method: Preliminary implications for improving field guarding. Hum.-Wildl. Interact..

[50] Hill C.M., Wallace G.E. (2012). Crop protection and conflict mitigation: Reducing the costs of living alongside non-human primates. Biodivers. Conserv..

[51] Distefano E. (2005). Human-Wildlife Conflict Worldwide: Collection of Case Studies, Analysis of Management Strategies and Good Practices.

