# Supplementary material for: Coexistence of Humans and Hamadryas Baboons in Al-Baha Region, Saudi Arabia—Emotional, Social, and Financial Aspects
Source: Animals (Basel). 2025 Dec 24;16(1):47. doi: 10.3390/ani16010047 (PMC12784782; doi:10.3390/ani16010047)
Supplement: Supplementary file 1 [file animals-16-00047-s001.zip › animals-3845478-supplementary.pdf]

supplementary material
online questionnaire

To serve the people and the related government authority in the region, this study aims to;

- **Explore the feelings of people toward having a non-human creature sharing their proximity**
- **Careful investigation of the role of people in the increased number of baboons and the possible unwelcome behaviour toward residents**
- **Exploring the ecological, social, positive, and negative impact of baboons on shared proximity**
- **Examine the possible causes of conflicts between people and baboons**
- **Hoping to reach the best solution, considering the experience of other nations**

NB: There are no indications that participation is mandatory or that there would be any sort of obligations

Demographic data:

1) gender
   - Female
   - Male

2) Age
   - < 30
   - 30-39
   - 40-49
   - >50

3) Education
   - None
   - Primary
   - Intermediate
   - Secondary
   - High education

4) Employment
   - Unemployed
   - Government employee
   - Private sector employee
   - Farmer

- Seller

5) Housing geography
   - Big city
   - Medium city
   - Small town
   - Village

6) Damaged property
   - Farm
   - Residence
   - Store
   - Others

Emotional aspect

1) I feel panicked and insecure
   - Agree
   - Somewhat agree
   - Disagree

2) I don't sleep deeply while I feel that the baboons are wandering in the area
   - Agree
   - Somewhat agree
   - Disagree

3) Monkeys are dangerous

   - Agree
   - Somewhat agree
   - Disagree

4) The presence of monkeys near homes and farms poses a psychological and physical danger to children
   - Agree
   - Somewhat agree
   - Disagree

5) I wish baboons would stay in my area

- Agree
- Somewhat agree
- Disagree

Social aspect

1) The presence of monkeys doesn't affect people's behavior and public system

- Agree
- Somewhat agree
- Disagree

2) The presence of monkeys affect the behavior of the local community negatively

- Agree
- Somewhat agree
- Disagree

3) The presence of monkeys is a negative phenomenon that deserve quick solutions

- Agree
- Somewhat agree
- Disagree

4) Conflicts and clashes between humans and baboons often occur when the monkeys are hungry

- Agree
- Somewhat agree
- Disagree

5) Monkeys attack people's property

- Agree

- Somewhat agree
- Disagree

6) People cannot enjoy the theme parks because Monleys control them

- Agree
- Somewhat agree
- Disagree

7) People use tools such as stones and sticks to keep monkeys off their property

- Agree
- Somewhat agree
- Disagree

8) I worry a lot about monkeys being near my property

- Agree
- Somewhat agree
- Disagree

9) Feeding monkeys in public places is a positive behavior

- Agree
- Somewhat agree
- Disagree

10) People can get injured when monkeys are near them

- Agree
- Somewhat agree
- Disagree

Environmental aspect

1) There are no cases of infection between humans from monkeys

- Agree
- Somewhat agree
- Disagree

2) Urban expansion has helped increase the number and presence of monkeys

- Agree
- Somewhat agree
- Disagree

3) Diseases are transmitted between monkeys and humans

- Agree
- Somewhat agree
- Disagree

4) monkeys contribute in environmental pollution

- Agree
- Somewhat agree
- Disagree

5) Damage occurs due to the increase in the number of monkeys

- Agree
- Somewhat agree
- Disagree

6) Monkeys cause damage to agriculture

- Agree
- Somewhat agree
- Disagree

Financial aspect

1) People incur losses on their property such as their transportation

   - Agree
   - Somewhat agree
   - Disagree

2) Monkeys contribute to damaging stores

   - Agree
   - Somewhat agree
   - Disagree

3) Monkeys cause damage to houses

   - Agree
   - Somewhat agree
   - Disagree

4) Monkeys cause damage to crops

   - Agree
   - Somewhat agree
   - Disagree

5) damages cost yearly (Riyal)

   - Less than 10000
   - From 10000 to 20000
   - More than 20000

6) Damage occurs

   - Weekly
   - Monthly
   - Yearly

Suggested solutions to control the presence of baboons

1) Controll monkeys reproduction

- Agree
- Somewhat agree
- Disagree

2) Preventing citizens from feeding monkeys

- Agree
- Somewhat agree
- Disagree

3) Install appropriate fencing to prevent monkeys from trespassing near properties

- Agree
- Somewhat agree
- Disagree

4) Develop environmental plans to reduce the presence of monkeys.

- Agree
- Somewhat agree
- Disagree

5) Enhancing public awareness of the negative impact of monkeys

- Agree
- Somewhat agree
- Disagree

6) Establishing regulatory frameworks to control monkey populations

- Agree
- Somewhat agree
- Disagree

7) Proper waste disposal reduces the spread of monkeys.

- Agree
- Somewhat agree
- Disagree
